# A Case of Secondary Leukemia Subsequent to Myelodysplastic Syndromes Successfully Treated with Azacitidine

**DOI:** 10.1155/2014/793928

**Published:** 2014-03-31

**Authors:** Takahiro Kumode, Ayano Fukui, Go Eguchi, Terufumi Yamaguchi, Yasuhiro Maeda

**Affiliations:** National Hospital Organization, Osaka Minami Medical Center, 2-1 Kidohigashi, Kawachinagano, Osaka 586-8521, Japan

## Abstract

Elderly patients with secondary acute myeloid leukemia (AML) following myelodysplastic syndrome (MDS) are often medically unfit for or resistant to chemotherapy, and their prognosis is dismal. In the present paper, we reported a case of secondary leukemia following MDS in an 80-year-old male patient who was deemed unfit for chemotherapy owing to his old age and poor physical condition. Despite a high tumor burden, treatment with AZA exerted a remarkable response, leading to an immediate cytoreduction in our case. Our results suggest that AZA can be an attractive therapeutic option for elderly MDS or AML patients, offering adequate efficacy and high tolerability.

## 1. Case and Introduction

**Figure 1:**
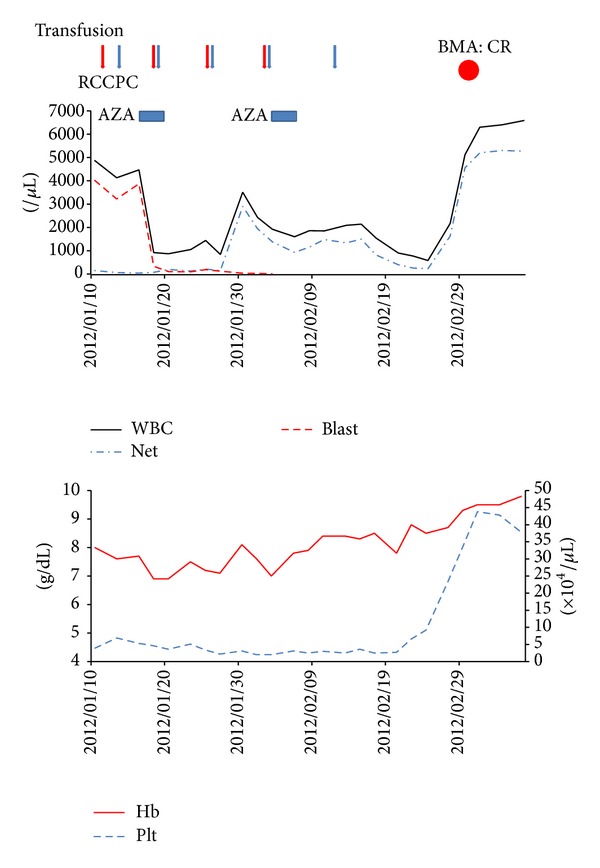
Clinical course after disease onset. RCC: red cell concentrates, PC: platelet concentrates, Aza: azacitidine, BMA: bone marrow aspiration, CR: complete remission, WBC: white blood cells, Net: neutrophils, Hb: hemoglobin, and Plt: platelets.

An 80-year-old Japanese man who was originally diagnosed with myelodysplastic syndromes (MDS) and refractory anemia with excess of blasts-2 in November 2011 was admitted to our hospital in December 2011 owing to the development of acute myeloid leukemia (AML). Prior to admission, the patient had refused therapy for MDS. His medical history included hypertension, chronic heart failure, chronic atrial fibrillation, and dementia. Laboratory findings at admission revealed a white blood cell (WBC) count of 3.3 × 10^9^/L with 85% blast cells, a hemoglobin (Hb) level of 7.5 g/dL, and a platelet count of 65 × 10^9^/L. Wilms' tumor gene (wt1) mRNA level in the peripheral blood was 12,0000 copies/*μ*g of RNA. Bone marrow aspiration showed 56.4% myoblasts, and G-band karyotype analysis revealed 46,XY,del(20)(q11.2q13.1) [13/20]. Surface marker analysis demonstrated that the blast cells were cluster of differentiations (CD)2−, CD7+, CD19−, CD13+, CD14−, CD15−, CD33−, CD41−, CD61−, CD64−, CD65−, CD11b−, CD34+, CD56−, CD117+, and human leukocyte antigen-death receptor+. The patient was deemed medically unfit for chemotherapy because of his old age and poor physical condition. Therefore, he was started on azacitidine (AZA) at 100 mg/day intravenously for 5 consecutive days every 4 weeks. As shown in Figure 1, after 2 cycles of AZA therapy, he achieved complete remission (CR) according to the Cheson criteria [1] despite of residual del(20)(q11.213.1) clone.

**Figure 2:**
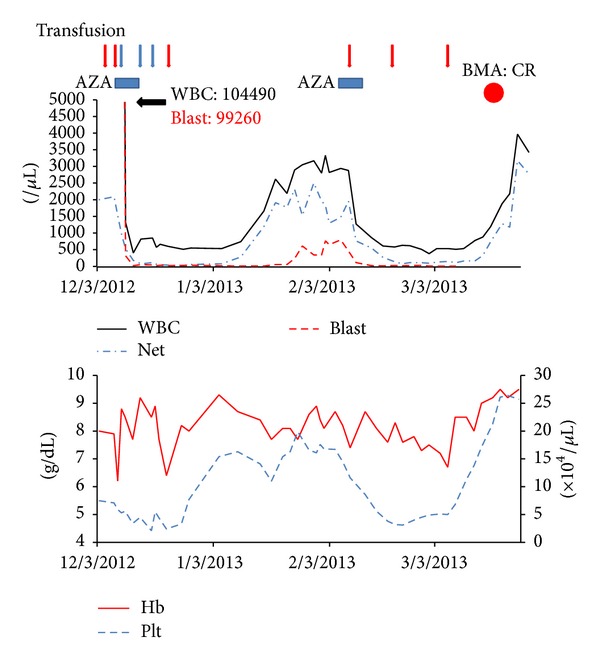
Clinical course after disease relapse.

The patient was able to maintain CR and a normal peripheral blood wt1 mRNA level without any AML therapy for almost a year. However, his disease relapsed with a high tumor burden, and he was readmitted to our hospital in November 2012 (Figure 2). Laboratory analysis of peripheral blood revealed a WBC count of 104.5 × 10^9^/L with 95% blast cells, an Hb level of 7.9 g/dL, and a platelet count of 71 × 10^9^/L. AZA therapy was once again initiated, resulting in a rapid reduction of peripheral blood blast cell percentage to <5% immediately after therapy started. After 2 cycles of AZA therapy, the bone marrow demonstrated persisting multilineage dysplasia with a reduction of blasts to <5%, indicating CR despite of residual del(20)(q11.213.1) clone. Although the patient continued to receive AZA therapy, he succumbed to disease progression in July 2013, which was 20 months after his original diagnosis of AML (Figure 2).

## 2. Discussion and Conclusion

Elderly patients with secondary AML following MDS are often medically unfit for or resistant to chemotherapy, and their prognosis is dismal. The Cancer and Leukemia Group B and AZA-001 studies have confirmed the efficacy of AZA in high-risk MDS patients whose bone marrow blasts were ≥20% but <30%, which currently meets the criteria for a diagnosis of AML according to the World Health Organization Guidelines [2, 3]. Subsequently, several other studies have suggested that AML patients could benefit from AZA therapy. In a study involving 82 AML patients, including 25 with secondary AML subsequent to MDS, Maurillo et al. reported that AZA was a potentially effective therapy for elderly patients who had previously untreated AML with a WBC count of <10 × 10^9^/L [4]. Al-Ali et al. studied 40 AML patients, including 20 newly diagnosed and 20 relapsed or refractory cases, who were deemed medically unfit for or resistant to chemotherapy. Among these patients, 16 had AML secondary to MDS. All enrolled patients were treated with AZA. Those with newly diagnosed AML experienced a median survival time of 7.7 months and an estimated 1-year survival of 39%, with a median follow-up period of 13 months. These reported results were superior to the published outcomes of standard nonintensive treatments. In addition, the median survival time of newly diagnosed AML patients who achieved stable disease was statistically similar to that of patients in CR, partial remission, or hematological improvement [5]. More recently, in a single-institutional study enrolling 227 consecutive older AML patients, including 74 with secondary AML, van der Helm et al. retrospectively compared the efficacy and tolerability of AZA, intensive chemotherapy, and best supportive care. They reported that in comparison to intensive chemotherapy, AZA resulted in a comparable overall survival but higher tolerability [6]. These observations suggest that, unlike intensive chemotherapy, achieving CR with AZA might not be necessary for an improved survival in elderly AML patients. Currently, no biological prognostic factors have been established to predict AZA's efficacy in MDS or AML patients. Itzykson et al. investigated the effect of the ten-eleven translocation 2(*TET2*) gene mutations on AZA's efficacy in 86 MDS or low blast count AML patients and reported that the presence of a* TET2* mutation predicted a higher response rate to AZA than the presence of wild-type* TET2 *did. However, response duration and survival were comparable between mutated and wild-type groups [7]. On the other hand, Voso et al. showed that* TET2* mutations did not have impact on the response to AZA and there were no differences of duration of response and survival between mutated and wild-type groups [8]. More recently, several researches about predictive markers in MDS after treatment of hypomethylating agents were reported [9–12]. According to some reports,* TP53* mutations did not predict response to AZA and their prognosis was identically dismal [9–11]. Although the patients group was small, patients with del20q might have higher response to AZA [9]. Actually, our present case had del20q as well and was seen as remarkable response to AZA. Ahn et al. reported that* SRSF2*, spliceosomal gene, mutation might be regarded as a risk factor of leukemic transformation compared with other spliceosomal gene mutations [12]. It has been suggested that the efficacy of AZA in AML patients with high tumor burden is limited and that cytoreductive chemotherapy is necessary for these patients. However, in the present case with a high tumor burden, AZA exerted a remarkable response, leading to an immediate cytoreduction similar to what is observed with other intense chemotherapy regimens. Therefore, our results suggest that there are good responders to AZA among both MDS and AML patients. If these good responders could be identified via useful biological prognostic factors prior to the beginning of therapy, treatment with AZA could be encouraged. In conclusion, AZA can be an attractive therapeutic option for elderly MDS or AML patients who are deemed medically unfit for or resistant to chemotherapy.
